# Navigating distrust and competing priorities during the COVID-19 pandemic: insights from Florida to strengthen cooperation

**DOI:** 10.3389/fpubh.2025.1686011

**Published:** 2025-11-18

**Authors:** Rachel N. Waldman, Anicca Liu, Johnathan H. Duff, Bonnie E. Deal, Jacob N. Batycki, Abhirami Sriganeshan, Ernesto A. Pretto, Jorge Saavedra, José Szapocznik

**Affiliations:** 1 Department of Public Health Sciences, Miller School of Medicine, University of Miami, Miami, FL, United States; 2 School of Communication, University of Miami, Coral Gables, FL, United States; 3 Department of Anesthesiology, Perioperative Medicine and Pain Management, Miller School of Medicine, University of Miami, Miami, FL, United States; 4 AHF Global Public Health Institute, Fort Lauderdale, FL, United States

**Keywords:** public health, COVID-19, distrust, competing priorities, cooperation, adherence, compliance, cultural competence

## Abstract

**Introduction:**

Stopping an infectious disease outbreak relies on a coordinated set of actions across public and private institutions and the wider public. However, cooperation with public health recommendations was notably hindered during the pandemic by widespread distrust in science and government and the notion that public health competed with other priorities. This study aims to examine the factors driving distrust and competing priorities in Florida, and potential pathways to overcome these issues.

**Methods:**

We conducted an additional analyses of qualitative data from our original study involving 25 semi-structured interviews with Florida stakeholders from government, academia, and the private sector. We employed a deductive-inductive approach to qualitative content analysis, using themes from the initial study as a guiding framework while allowing for the emergence of additional insights.

**Results:**

Interviews revealed that inadequate transparency and data availability, politicization, and poor communication were perceived as undermining public trust in science and decision-makers during COVID-19. The economy and individual rights were discussed as priorities competing against public health during the pandemic. Objectives for building trust and balancing priorities included five essential areas: transparency, representation, communication, education, and balance.

**Discussion:**

These challenges and objectives reflect the need for a reimagined approach to public health policy and practice—one that is rooted in trust and respect for diverse value systems. By leveraging core collective values that cut across political ideologies, we may mitigate polarization and perceived stigmatization to build a more culturally resonant public health practice.

## 1 Introduction

What constitutes “success” when responding to a pandemic? While preventing disease and loss of life are often the first considerations, the COVID-19 pandemic revealed the complexity of this question. Concerns around trust, the economy, and personal autonomy shaped how many Americans experienced the pandemic and how policymakers responded to it. Without agreement on what a successful pandemic response entails, U.S. states varied in how closely their policies aligned with public health recommendations, resulting in a discordant national response (1).

Florida’s pandemic approach, for example, often diverged from public health guidance and was primarily influenced by the priorities set by the governor (2, 3). The state response entailed relatively fewer restrictions on movement and businesses and a swift rollback of pandemic policies beginning May 2020 (3, 4). Additionally, Florida was one of the few states that refrained from instituting a mask mandate and received national attention after advising against COVID-19 vaccination for children (5, 6). Notably, Florida’s public health response became fully centralized during the pandemic, with decision-making concentrated at the state executive level (2). Through Emergency Order 21-102, the Florida Governor curtailed the authority of local governments aiming to block policies that might restrict the “presumption of commercial operation and individual liberty” (7). Further, in the midst of the pandemic, the State Surgeon General was succeeded by an appointee who more closely echoed the governor’s positions, particularly in expressing greater hesitancy toward vaccines (8). These actions distinguished Florida from other states that better aligned with public health recommendations and allowed local government and public health officials a greater role in determining responses. Among U.S. states, Florida ranked 8th in COVID-19 cases and 12th in COVID-19 deaths per 100,000 people (9)—outcomes likely influenced by the state’s policy responses, as well as other factors, such as population demographics, mobility patterns, and healthcare capacity (10, 11).

There was also considerable disagreement among citizens regarding the right course of action during the pandemic, resulting in varying uptake of public health guidance. Since government and public health officials serve as primary conduits for public health communications and recommendations, the extent of public trust in these leaders—or lack thereof—can profoundly shape cooperation. In this context, trust refers specifically to vertical trust, which encompasses confidence in institutions, such as government and public health agencies (12), and the expectation that their intentions and actions reflect the public’s best interests (13). Suhay et al. found that trust in state government and local health officials was associated with higher levels of engagement in protective health behaviors, such as mask wearing and social distancing during COVID-19 (14). Conversely, trust in the federal government during the initial stages of the pandemic was associated with lower adherence to these public health protections (14). These findings allude to how broader partisanship dynamics may reinforce or undermine the credibility of public health recommendations. Beyond the messenger, effective communication of pandemic-related messages has been identified as a key factor to promoting adherence to public health efforts (15, 16). During the pandemic, however, inconsistent messaging, frequently evolving recommendations, and politicized rhetoric, contributed to skepticism, ultimately complicating adoption of public health guidance (17).

While the temporary suspension of normal activities is necessary to limit viral spread, concerns about potential trade-offs, such as economic stability and personal autonomy, resulted in mixed adherence to public health recommendations (18). These concerns shaped the narrative that other priorities were in direct competition with public health. Competing priorities were invoked by some decision-makers to advocate against implementation or continuation of pandemic restrictions, hampering disease mitigation efforts (16). For instance, the Florida Governor claimed the state’s relatively relaxed policy approach “kept our state open and free,” unlike states that instituted stricter restrictions on movement during the pandemic (19). Among the public, some interpreted directives to wear masks and to practice social distancing as an infringement on their personal liberties (20).

Others viewed the pandemic response as a significant source of economic disruptions, also fueling opposition to public health guidelines (21). Concerns around the impact of public health measures on the economy were not unfounded. Walmsley et al. found that the largest real GDP losses in Florida during COVID-19 were associated with mandatory businesses closures and the gradual easing of restrictions (22). Despite Florida’s relatively relaxed COVID-19 policy approach (3), 85.1% of the state’s small businesses reported a loss of revenue during the first year of the pandemic, with some owners expressing additional need for financial support to remain operational (23).

The difficulties in rallying the American public and institutions around shared goals during the pandemic underscore a key failure in cooperation. There is a need to deeply understand the tensions around distrust and competing priorities to inform a more united front in responding to future health emergencies. Florida is an important case study, representing a political landscape shaped by a Republican-majority state government and conservative-leaning public. The state’s approach to the pandemic exemplified how competing priorities and distrust in public health can shape public health policy decisions and public adherence, offering valuable lessons for future pandemic responses. Moreover, recent events, such as Florida’s decision to end childhood vaccine mandates (24), demonstrate a continuing trend of health policies that prioritize individual autonomy over collective wellbeing—a trend that has persisted beyond the COVID-19 pandemic. If public health fails to garner more support from decision-makers and the public, this decision to forgo mandatory vaccinations may set a precedent for other states, with wider consequences for infectious disease control. The State of Florida, therefore, offers critical insights relevant to an increasingly polarized population.

This stakeholder-informed qualitative study gathers perspectives from leaders involved in Florida’s COVID-19 response to expand on the specific tensions impacting cooperation during the pandemic. Stakeholders from government, academia, and the private sector discussed how distrust and competing priorities challenged a united approach to combatting COVID-19 in Florida, in addition to offering strategic approaches appealing to the broader public. In response to growing calls for greater integration of social science perspectives within the public health discipline (25), we draw on theories of stigma (26) and national identity (27, 28) in our discussion of polarization fomenting resistance to public health efforts. From these perspectives, we explore how core collective values—those that cut across political ideologies— might be leveraged to shape a more culturally resonant public health practice.

## 2 Materials and methods

The findings presented here constitute one component of a larger qualitative study (16), which aimed to understand how the State of Florida could improve future pandemic preparedness and response (PPR) in light of the COVID-19 pandemic. During the initial phase of the study, lack of cooperation with public health recommendations around disease mitigation, as it related to American culture, emerged as a significant topic across interviews. To better understand this pattern, we conducted an additional analysis to explore the challenges around cooperation in greater depth.

### 2.1 Participants

This study used qualitative methods to gather perspectives on PPR from stakeholders in government, academia, and the private sector in Florida (See Table 1). Participants were recruited from January 25, 2021, to December 7, 2022. The initial sample targeted Florida Department of Health (FDOH) County Directors (representing all 67 Florida counties) and was later expanded to include current and former elected officials, experts in academia, and private sector professionals involved in disaster response and the hospitality industry in Florida. Recruitment and data collection have been reported in greater detail in a previous publication (16).

**Table 1:** Participant affiliation by sector.

Sector	Subsector/affiliation	Gender		Total
		M	F	
Government	Florida Department of Health (state and county level)	5	8	13
	Florida Division of Emergency Management/Federal Emergency Management Agency	2	0	2
	Florida Legislature	3	1	4
Academia	Universities	1	2	3
Private sector	Disaster Management	1	1	2
	Hospitality	2	1	3
Total		14	13	27

### 2.2 Interviews

Semi-structured interviews were used to collect participants’ insights on PPR in Florida. Participants provided verbal consent to be recorded prior to the interview and all were notified of the confidentiality of their responses. Twenty-five interviews were conducted with 27 participants (2 group interviews and 23 individual interviews) on a video conferencing platform between January 2021 and December 2022. Of the 25 interviews included in analysis, 22 were recorded and transcribed by a professional transcription service; detailed notes were taken for the remaining 3 interviews. All data were stored in a secure server and were anonymized via participant ID numbers. The University of Miami Institutional Review Board (IRB) determined that the study was exempt from full IRB review.

### 2.3 Analysis

A comprehensive presentation of the methods and results from the full study have been reported previously (16). The current analysis builds on the initial study, in which lack of cooperation with public health guidance emerged as a major theme, offering lessons for future public health responses. For this analysis, we employed a deductive-inductive approach to qualitative content analysis (29). This approach takes into account the initial study while facilitating the emergence of new insights. We reviewed codes from the initial study to evaluate their relevance to our focus on issues related to cooperation with public health guidelines for disease mitigation. Through discussions, we highlighted one subcategory from the initial analysis, *culture of public health*, which encompassed themes of *trust* and *prioritization of public health*. The current analysis centers on these two topics because, while the other subcategories emphasized areas that could be reformed materially (i.e., laboratory capacities, workforce, and communications), *trust* and *prioritization of public health* encompassed perspectives on influencing behavior to strengthen adherence to public health. However, we noticed that cultural dimensions central to cooperation cut across other subcategories and topics, warranting a deeper analysis of trust and prioritization of public health through a reexamination and recoding of all data.

In the deductive phase of our present analysis, we utilized an *a priori* framework of two main challenges: *distrust* and *competing priorities*, which are adapted from the names of the original themes to correspond directly with the drivers of disharmony in cooperation around disease mitigation efforts. Recognizing that relevant material could be found beyond what was originally coded under these themes, we applied them to the full dataset. Two analysts (RW & AL) independently examined the transcripts and notes, assigning quotes to one of the two challenges.

Shifting to an inductive approach, we took interpretive notes for each quote and noted, where relevant, objectives aimed at addressing identified challenges, which informed the development of the new coding framework. Challenges of *distrust* and *competing priorities* were analyzed first, and through iterative discussions, the coders identified a set of contributing factors for each theme. Additionally, five cross-cutting objectives needed to overcome the challenges were identified. The research question evolved inductively as the new coding framework was shaped through the analysis. Throughout each stage, the two coders consistently convened to review and refine the evolving framework, compare interpretations, and resolve any differences in analysis.

## 3 Results

### 3.1 Challenges

During interviews, participants highlighted challenges and potential avenues around fostering adherence to public health recommendations and cultivating a sociocultural context that promotes cooperation. We first present the challenges of *distrust* and *competing priorities*, along with factors that participants reported as contributing to each of these themes (Table 2). For *distrust*, three contributing factors emerged: *data availability and transparency*, *politicization*, and *communication*. Under *competing priorities*, two factors were identified: *the economy* and *individual rights*.

**Table 2:** Factors contributing to distrust and competing priorities during COVID-19.

Challenges	Contributing factors
Distrust	Data availability and transparency Limited and evolving information of a novel disease Purposeful restriction and misrepresentation of data
	Politicization Pre-existing polarized landscape Differentiation of policy implementation along party lines Censorship and sidelining of public officials that opposed the state government’s stance
	Communication Poor delivery of messaging Misinformation from the media and decision-makers Absence of a universally trusted communicator
Competing priorities	Economy Florida’s reliance on its hospitality industry Perceived differences between public health and the interests of business owners Hardships of service industry workers
	Individual rights Importance of autonomy and preservation of personal freedoms Discrepancies between American values and public health recommendations

#### 3.1.1 Distrust

##### 3.1.1.1 Data availability and transparency

Public trust was undermined by limited access to reliable information, in part a consequence of COVID-19 being a novel disease. Early in the pandemic, public health decisions were made with “an abundance of caution” (member of Florida Legislature) given the minimal information available on how the virus spread and its potential severity. Some participants from the hospitality industry and Florida Legislature believed decisions based on limited information were ultimately harmful and negatively affected the credibility of public health. One participant from the Florida Legislature perceived the early implementation of stringent policies damaging to the reputation of public health and its proponents, lauding Florida’s decision to avoid maintaining its restrictions for too long:

I think the public health community really hurt itself during this pandemic…out of an abundance of caution. We didn't know any better, but the general public… think the public health community is full of crap now… Florida didn't [maintain restrictions] and Florida was fine…There are people who died, and we don't want to minimize that…But we quickly realized it was those who were compromised [who died]. Everybody else was fine. (member of Florida Legislature)

Others from Florida Department of Health (FDOH), however, found the precautionary approach appropriate under the uncertainty of the early phases of the pandemic.

At the onset of the pandemic, public health recommendations evolved rapidly in response to emerging information about the disease. Participants from academia, Florida Division of Emergency Management (FDEM), FDOH, and the hospitality industry stressed that the shifting guidance fostered distrust. An FDEM leader asserted that “when government is communicating during an emergency, you have to be right,” noting that the CDC’s inconsistency on guidance to wear masks exacerbated pre-existing doubts. Participants from FDEM and the hospitality industry suggested that when public health recommendations wavered without clear explanation, confidence in public health leaders waned. Inconsistencies were interpreted as a lack of certainty on the effectiveness of recommended interventions:

…if there is already doubt out there and then the government equivocates and then feeds into that doubt…[it] makes people believe that their original position was correct. For the folks that didn't believe masks worked…when the CDC made that change, it solidified that position…(FDEM leader)

Participants from FDOH, FDEM, and the Florida Legislature also spoke to problems with data transparency, including the state’s purposeful restriction of public access to information.

Referencing Florida’s efforts early in the pandemic, a county FDOH Director discussed how the state “started off very transparent” through the creation of a COVID-19 dashboard that provided daily updates on outcomes. Another participant from FDOH at the state level recounted how in April 2020, Florida was “ahead of the curve” and received praise for being “what every state was told to strive to be.” The COVID-19 dashboard was later removed from the FDOH website, with Florida referred to as the “first state to stop reporting” (state-level FDOH leader). Without adequate data reporting, public health professionals faced difficulties establishing the public’s trust in protective public health measures:

…in the State of Florida…it's not possible for anybody outside of [FDOH] to gain access to the detailed case data that would allow us to test some of the hypotheses [about] various interventions…(academic)

The removal of the COVID-19 dashboard was regarded as “harmful to the community” (county FDOH Director) and left individuals with “no information and no agency to make informed decisions about their own health, their children’s health, [or] the relative safety of going out in public” (state-level FDOH leader). It also cultivated “suspicion about the data” (academic), motivating concerns that the state health department purposefully hid data and deliberately refused media requests for information. One state-level FDOH leader suggested that the removal of public access to data was often justified by “this idea that people do not have a right to know or that they will not understand.” This sentiment sparked contention within FDOH, where debates intensified over how much information should be disclosed to the public. At the county level, several local health directors suggested that their hands were tied by the state but expressed support for maintaining the dashboard.

##### 3.1.1.2 Politicization

Several participants from the Florida Legislature, FDOH, and FDEM discussed how trust in public health was also shaped by the growing polarization between the nation’s two major political parties. They believed that this tense political climate permeated COVID-related attitudes, knowledge, and behaviors of both decision-makers and the public. Referring to this underlying polarization, a member of the Florida Legislature described how the ‘us’ versus ‘them’ mentality has historically guided the formation of alliances and oppositions across political parties and government agencies. This participant spoke to how partisanship inevitably created allegiances during the pandemic:

You’re on one side or the other… So under what administration is this pandemic and who are we going after? And so, what federal agency am I supposed to trust on this stuff anymore? (member of Florida Legislature)

Another member of the Florida Legislature added that while antagonism between the Republican Party and the CDC existed before COVID-19, it escalated during the pandemic, with Republicans “seeking to invalidate” the CDC and, by extension, other public health institutions.

Participants from FDEM, FDOH, and the Florida Legislature believed that trust in public health agencies was divided along party lines, shaping “two realities” (FDEM leader) around COVID-19. They described how one segment of the population believed in the severity of the crisis, while the other denied such gravity. As observed by one participant from FDEM, such public skepticism had never presented in other emergencies—namely hurricanes—in the state:

When a hurricane hits, you don’t have half the population saying it was a [category] five and the other half of the population saying, no, it was a category two hurricane…But in this emergency, there were two sets of facts, sometimes more. When half the population is not willing to accept the reality of what is happening…that is going to be a tremendous challenge. (FDEM leader)

One Florida Legislator suggested that polarization generated pressure for individuals to conform to the views on COVID-19 endorsed by their respective political party. The result of this was a public divided around two sets of ‘facts’—not only about the virus, but also the interventions and measures used to respond to the pandemic, such as masks, social distancing, and vaccines.

Conflicting state-level pandemic responses, driven by partisanship, was believed to contribute to distrust around public health interventions. It was noted by a few participants that Florida followed the pattern of Republican-led states, generally entailing fewer restrictions and recommendations consistent with CDC guidelines than in Democrat-led states. Variations in public policy response along party lines prompted suspicions from the public about whether decisions were made “based on good information [or] political philosophy” (hospitality industry leader). Participants raised concerns from both ends of the political spectrum. Some from academia and FDOH felt that many states with Republican governors (including Florida) had purposefully rejected evidence-based interventions out of political interests. Meanwhile, others from the Florida Legislature and hospitality industry believed that states with Democratic governors implemented excessive COVID-19 policies as political retaliation.

Several participants from academia and FDOH at the state and county levels discussed Florida’s COVID-19 vaccination stance as an example of politically motivated decision-making that stoked distrust. One participant from FDOH claimed that the Florida Governor’s position on COVID-19 vaccines shifted after President Biden enthusiastically promoted them in March of 2021, following a contentious election that further divided the country on the pandemic:

Before [March 2021], you can think of [Florida’s governor] flying all over the states going to all these different vaccine events. Didn’t happen after that. There was no further talk from the governor about having people getting vaccinated. And it wasn’t just [Florida’s governor], it was the other red state governors. (state-level FDOH leader)

This participant, among others from FDOH and academia, was alarmed that Florida’s advisory against COVID-19 vaccines for children contradicted recommendations from the CDC, validating anti-science positions among the governor’s Republican supporters. Further, one FDOH leader stressed that the promotion of individuals with ‘antivaccine’ views into positions of power, namely that of the Florida Surgeon General, was a tactic catering to the state’s conservative majority and allowed these attitudes to proliferate among the public.

Speaking to political will, participants acknowledged the difficult position government and public health officials faced when making decisions and publicly expressing perspectives on public health interventions, citing potential fears of jeopardizing their position or relationships with their political base. Specifically, a state-level FDOH leader referenced an incident in which a former State Surgeon General was prohibited from public communications after voicing a perspective that contradicted the position of the governor:

[Our former Surgeon General] suggested in a meeting, I believe it was in March of 2020, that we might be needing to wear masks into 2021. And he was aggressively pulled out of that meeting on camera and escorted out and he was never allowed to participate in public meetings after that point. He really was trying to do the best job that he could do given the circumstances...(state-level FDOH leader)

One participant from the Florida Legislature questioned how the public could trust the government given its censorship of public health officials. Expressing dissatisfaction with the silencing of the former Surgeon General they stated, “the idea that we were prohibited from asking our top public health official in our state questions in the middle of a pandemic was beyond frustrating.” They asserted that the removal and sidelining of public health officials likely discouraged decision-makers from opposing the state’s stance, thereby fueling public distrust.

##### 3.1.1.3 Communication

Participants across all sectors emphasized how inconsistent, misleading, and politicized messaging undermined public trust in science and decision-makers during the pandemic, and in turn, impacted adherence to public health recommendations. Specifically, concerns were raised around the delivery, content, and source of pandemic-related messages to the public. Several participants felt that the government and mainstream media communicated “in ways that were not trustworthy” (academic) by presenting information in a negative, fear-mongering tone that conveyed a “visceral vibe” (hospitality industry leader) rather than calm and measured discourse. They believed the content of messages also failed to inspire trust among the public, partly due to concerns around potential misinformation.

However, perspectives varied around what constituted misinformation and who was responsible. For instance, a few leaders from the Florida Legislature and hospitality industry felt that misinformation had been evident in the media’s ‘exaggeration’ of the pandemic, which they believed amplified public panic. Others from FDOH, emergency management, and academia, suggested decision-makers had contributed to misinformation by contradicting recommendations of public health institutions, or potentially manipulating data to suit political narratives.

Participants lacked consensus regarding who should lead communications in the event of a future pandemic. This pointed to a major issue surrounding messaging during COVID-19—there was no single public figure that was universally accepted and trusted as the authority on how to respond. In discussing future pandemic responses, some participants from FDOH cautioned against allowing government officials to take on the role of messenger. One argued that the public perceived such communications through a political lens due to prevailing “political tribalism” (state-level FDOH leader). Another participant discussed confirmation bias and a myriad of conflicting credentialed voices as affecting assessments of the trustworthiness of information:

People tend to follow the people that think like they do. And so, you’ve got folks on one side who say, “Listen, I’ve got a ton of [post-nominals] behind my name and so I know exactly what I’m talking about.” And then you’ve got somebody else on the other side who has just as many [post-nominals], if not more, saying the other thing. And you go, “I want to follow this guy because I like that better.” Well, that doesn’t help… people have lost trust, because there’s so many different opinions...(member of Florida Legislature)

The lack of a trustworthy messenger, therefore, allowed many individuals to remain entrenched in their existing views and to adapt recommendations according to pre-existing preferences.

#### 3.1.2 Competing priorities

##### 3.1.2.1 Economy

During interviews, some participants perceived the goals of public health—to minimize the spread of the virus and to preserve life—to be at odds with maintaining the economic system that Floridians rely on for their livelihoods. Tensions between the private sector and public health gradually became evident as the pandemic progressed, with a hospitality industry leader referring to the perspectives of both groups as “diametrically opposed.” It was said that industries central to Florida’s economy—hotels, airlines, cruise ships, resorts—believed their operations and ultimately, survival, were jeopardized by public health restrictions. Consequently, participants described a resistance to public health guidelines from the business community that grew as the pandemic continued.

Participants noted that pushback from the hospitality sector—one of the most consequential industries in the state—strained decision-making for public health. Political leaders, subject to the petitions of their constituencies, found decisions about the continued use of pandemic response strategies more complicated due to opposition from powerful constituents. A state-level FDOH leader described the way these pressures reached state leadership:

The number one industry in Florida is tourism and recreation. So, if all the bar owners, businesses, [and] resorts [owners are] saying, “you’re killing us, we’re losing money”—and these individuals are big donors to the administration—all of a sudden that puts a conflict in place [with public] health...”

Echoing these concerns, another participant from the Florida Legislature recounted that a pandemic committee hearing they attended was dominated by discussion of liability protection for businesses rather than strategies to mitigate the spread of COVID-19. As an elected official, this participant conveyed disappointment in the sway the business community possessed in shaping political agendas, especially amid a public health emergency.

Several leaders from FDOH and FDEM described an inherent challenge in persuading businesses to act against what they believed to be their best interests. As these participants pointed out, it was easier to appeal to larger corporations that have the ability to absorb losses. On the other hand, small businesses operated within thinner profit margins and were more prone to experiencing short-term disruptions as cataclysmic. One participant discussed the disproportionate willingness to cooperate with public health guidelines as tied with economic privileges:

Think about it, well, before [the Florida governor] did anything, who shut down voluntarily? It was Disney, it's Universal…That's not the problem. The problem are small local venues that die [during closures]…(FDEM leader)

Hospitality leaders spoke to the economic consequences of public health policies in relation to business preservation and the pressures they created for employers. One described the dilemma many employers faced early in the pandemic: deciding between allowing their small business to perish by prioritizing public health or defying restrictions and risking the health of their patrons and employees.

Participants noted that pandemic restrictions also exacerbated economic disparities among individuals, resulting in varying levels of willingness to comply with public health recommendations. Just as larger corporations could withstand a temporary financial slowdown better than small businesses, so could wealthier individuals compared to those with less means:

[The] whole system of, ‘let’s close everything down, send everybody home’ [is] just fine if you have some money in the bank and you’re good to go, but if you’re struggling paycheck to paycheck, it…put people through a lot of hardship (hospitality industry leader)

One participant described the difficulties faced by workers when choosing between continued financial earning and adhering to pandemic mitigation efforts:

We know that individuals, although they were sick, still went to work or were reluctant to test, and we understand why, because individuals need to work in order to make money to pay [for rent and food]…But unfortunately, what happens is that they went [to work] and they spread the virus to other individuals...(county FDOH Director)

Those unable to forgo a paycheck often felt the pressure to continue working under conditions potentially dangerous to their health and that of others, thereby prioritizing their urgent economic needs over health. One hospitality industry leader implied that individuals should have never been forced to grapple with such difficult decisions. These choices, they claimed, were evidence that the government had failed to ensure necessary protections were in place.

##### 3.1.2.2 Individual rights

Participants across all included sectors also spoke to the prominence of individual rights, a core American value, which conflicted with the more collectivistic approaches espoused by public health. Akin to earlier discussion on the economy, participants noted that many viewed public health measures as a barrier to exercising individual freedoms. Several participants attributed the prioritization of personal liberties over collective wellbeing during the pandemic to be a product of U.S. culture. Put simply, a member of the Florida Legislature stated, “If you learn anything or I learn anything about my fellow Americans, they still do not like being told what to do, even when it’s the right thing.” This participant, among others, implied that even when the public trusted in the science, many individuals were still unwilling to comply with recommendations because they valued autonomy and were reluctant to receive external directives.

A few participants stressed how priorities around personal freedoms resulted in poor execution of public health measures. The measures implemented by state governments to mitigate the spread of COVID-19, one state legislator explained, were often met with resistance by a public who perceived these interventions to be an inconvenience to their everyday lives. One FDOH leader, for example, recounted how some individuals refused to answer calls from contact tracers or provide personal information to avoid quarantine. An academic added that political tensions likely contributed to this evasion of contact tracers, as many individuals expressed concerns over providing personal information to the government or a public health agency whom they did not trust to protect their privacy:

The problem was that…[we were not able to reach even] half the people, because the people weren't picking up their phone, or if they did pick up their phone, they weren't willing to talk to the health department…Contact tracing has been really important in public health in infection control…And I think, for public health, this is a remaining challenge, how do we get people to cooperate? How are they going to trust that their information will be confidential…?

Another participant suggested that the U.S. would likely struggle to enforce quarantine mandates because its culture strongly upholds individual liberties:

… different countries do different things and certainly in a police state you can do [quarantine] very effectively, we have a little more trouble with that here. I’m perfectly honest with you, I don’t think we could quarantine people right now if we wanted to. (county FDOH Director)

This participant, as well as others from the Florida Legislature and FDEM, alluded to U.S. culture as unlike those of other countries, impacting its pandemic response. They suggested that since collectivist interventions require behavioral changes that give precedence to the health of the community over the rights of the individual, they were incompatible with American culture. A member of the Florida Legislature felt concerned about the impact of “Covid fatigue” on future pandemic responses, citing a rising sense of burnout and lack of willingness among the public to tolerate the compromises associated with pandemic restrictions.

### 3.2 Objectives to overcome challenges

**Figure 1:**
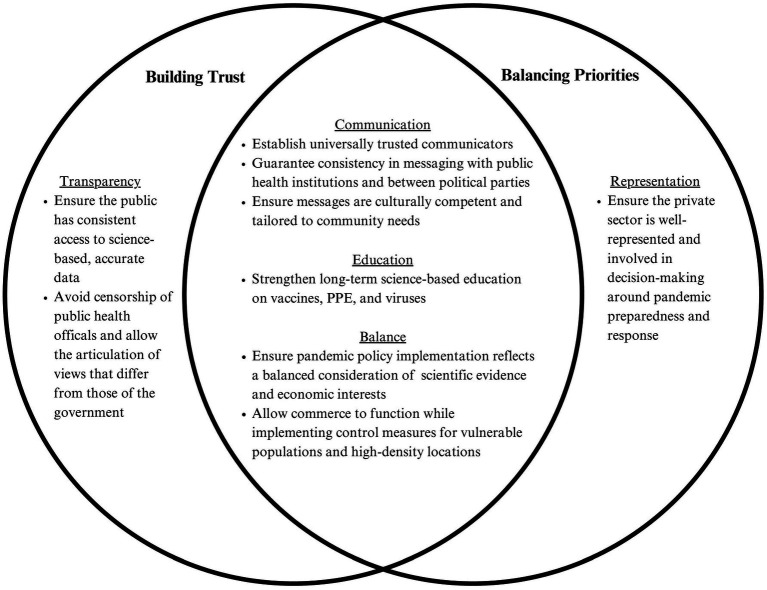
Objectives to overcome distrust and competing priorities.

While reflecting on the challenges of distrust and competing priorities, participants proposed objectives aimed at fostering trust and balancing priorities. These objectives reflect the fundamental components necessary to addressing these challenges. Participants identified five core objectives: *transparency, representation, communication, education,* and *balance*. Notably, several of these objectives were discussed in the context of both distrust and competing priorities, underscoring the interconnectedness of these two issues (see Figure 1).

#### 3.2.1 Transparency

Participants suggested two areas for improvement that would strengthen transparency, and therefore, help foster a greater sense of trust in public health. First, participants emphasized the need for consistent data transparency through the distribution of apolitical, evidence-based information that has been validated by a trusted source. Open access to reliable data was said to promote trust in science and encourage informed decision-making by the public. Participants believed that maintenance of the Florida COVID-19 dashboard, which was revered for providing comprehensive epidemiological data, exemplified the kind of transparency that would cultivate trust in state-sourced information.

Additionally, participants discussed achieving transparency through avoiding censorship of public health officials and enabling the expression of perspectives oppositional to those of the governments. A state-level FDOH leader recommended “do not fire your experts” to prevent further erosion of trust in public health officials. This participant suggested that especially in Republican-led states, allowing public health authorities to carry out their public health duties, even when they oppose the governors’ views, could reinforce the credibility of these officials.

#### 3.2.2 Representation

Several participants identified the need for broader representation of the private sector in decision-making around PPR. Participants argued that doing so is necessary to balance interests and prevent incongruence between economic activity and mitigation efforts. This balance might create resonance with the public and allow individuals to recognize the value of public health without perceiving it as a threat to their values and needs. Participants also noted that increased private sector representation generates buy-in from business owners to participate in PPR and builds political will among decision-makers otherwise hesitant to induce economic repercussions.

#### 3.2.3 Communication

Participants proposed improving communication, including the delivery, content, and source of pandemic-related messaging. Regarding message delivery, participants highlighted the need to approach communication with a calm, rather than sensationalist, demeanor to avoid skepticism. Recounting successful experiences, one county FDOH Director emphasized the need for culturally competent communication—defined here as tailoring messages to the specific needs of marginalized communities and delivering them in the appropriate language and through widely used and trusted platforms. Participants expressed opposing perspectives on how to motivate a public that values individual rights. Some suggested using forceful language to prevent misinterpretation of the message. Others recommended a more relaxed communication style that emphasizes freedom to accept or disregard messaging to avoid perceptions that public health comes at the expense of personal liberties.

On message content, participants discussed the need for consistency between political parties, decision-makers and public health institutions, as well as the importance of highlighting positive trends. Rather than having a communications environment filled with opposing messages, they stressed that disagreements must be handled behind closed doors to prevent polarization. Additionally, to boost morale, a few believed messaging could highlight successes, such as the number of individuals who were tested, treated, and vaccinated. Positive progress could be presented alongside information on hospitalizations and mortality, which, while crucial to keep a public informed about the state of an outbreak, could be discouraging and stoke fear if presented alone. Also, although a seemingly unsurmountable notion in current times, participants underscored the need for universally trusted communicators to deliver messages to the public. As previously mentioned, participants were not in agreement regarding who should take this role. Rather, they pointed to the need for an ‘apolitical’ communicator who can speak in a balanced manner to diverse values and concerns.

#### 3.2.4 Education

Participants identified improving formal education as a key objective that would increase scientific literacy and promote trust in public health. Several participants discussed the need for enhancing long-term, science-based education from elementary to post-secondary education. Specifically, participants advocated for better knowledge about viruses, bacteria, vaccinations and non-pharmaceutical interventions. Although there was broad agreement on the need to strengthen education, participants diverged in their rationales for why such improvement was necessary. Some participants viewed education as a tool for enhancing individual decision-making, whereas others emphasized scientific education as a means to normalize a collective health orientation. For example, a participant from the latter called for embedding scientific understanding “into the cultural fabric of society” (member of Florida Legislature) to empower future generations to adopt protective behaviors. Meanwhile, the others suggested that improving education would allow individuals to make informed choices on their own terms—potentially reducing the need for restrictive public health directives.

#### 3.2.5 Balance

The final objective discussed by participants centered on developing a more balanced pandemic response to achieve greater harmony between public health, economic needs, and individual rights, resulting in strategies that could be embraced by all. Participants stressed that a balanced approach should make use of the best evidence available while considering economic realities and core social values, such as personal liberties. For some, this entailed allowing commerce to function as much as possible while implementing control measures for vulnerable populations and high-density locations. Participants also suggested that a harmonious approach addressing diverse needs could help cultivate greater trust in decision-makers, improving uptake of recommendations, especially among individuals concerned about stringent measures as a form of political oppression.

## 4 Discussion

Effectively combatting infectious disease outbreaks requires a high degree of cooperation, characterized by coordinated actions among all citizens and organizations. Our study aimed to explore the underlying tensions driving distrust and competing priorities in Florida and to identify ways to strengthen cooperation between public health, government, the private sector, and a divided public. Through interviews with Florida stakeholders, we found that lack of transparency, politicization, and unclear and inconsistent communication during COVID-19 contributed to increased skepticism toward public health. Further, two interests were positioned as trade-offs to public health during the pandemic, namely the economy and individual rights. To overcome distrust and competing priorities, participants outlined five broad objectives— transparency, representation, communication, education, and balance—intended to guide future public health approaches. While the diversity of participants precluded full consensus on how to actualize specific recommendations, these objectives signify broad areas of agreement aimed at enhancing cooperation. Working toward these areas requires sustained and concerted efforts to create a new public health paradigm, one defined by a foundation of trust and a more nuanced understanding of value systems.

Our study highlights the erosion of public health’s credibility during the pandemic in Florida, contributing to a context of distrust in public health and science. However, such skepticism did not emerge with the novel virus. Rather, the COVID-19 pandemic developed in a landscape where public trust in scientific and medical guidance was already fragile due, in part, to ideological differences (30). Although some conservative leaders previously championed major public health initiatives such as PEPFAR (31), Oreskes and Conway have argued that modern conservative distrust in public health can be traced to anti-government attitudes that were amplified during the Reagan administration (32). In the 1980’s, some conservative leaders challenged scientific findings on environmental issues, such as ozone depletion, which implied a need for increased government regulation of environmental practices (32, 33). In recent years, these antiregulatory attitudes have expanded to target infectious disease responses, an area of public health often perceived as threatening individual freedoms through mandates and restrictions on movement (31). Concerns around public health’s promotion of government overreach may partly explain the hyper partisan division of vaccination policy and the rise in vaccine hesitancy among political conservatives over the past few decades (34, 35). The evolution of these attitudes demonstrate the interconnectedness of distrust and competing priorities—when individual freedoms take priority over collective benefit, government intervention may be taken to be excessive, contributing to and reinforcing distrust in public health. Among other Americans, distrust has also been historically fueled by scrutiny of the profit motive in health care (36, 37) and the history of unethical medical experimentation (38)—factors beyond the scope of our study.

Opposition to public health and science, however, is not solely a product of contemporary conservative political ideology; rather, it is partially rooted in feelings of exclusion and social division, which public health institutions have sometimes exacerbated. Stigma theory offers a valuable framework for understanding how public health can better engage with audiences that feel alienated (26). In the U.S., a growing pattern of perceived stigmatization has emerged, with some of today’s conservatives feeling politically sidelined, particularly by the media, academia, and other liberal institutions (39). During the pandemic, non-compliance to public health recommendations were often framed by public health officials and their proponents as disregard for the wellbeing of others, carrying strong moral righteousness (40). This likely contributed to the formation and reinforcement of stigmatized identity for those who resisted public health restrictions and calls to vaccinate. Groups deemed deviant may embrace stigmatized identity as a source of pride (26). Rather than encouraging adoption of norms, messages with undertones of shaming can instead reinforce conservatives’ identification with the labels imposed on them (i.e., anti-vaxx and anti-science) and produce a boomerang effect (41), resulting in non-compliance with public health guidance. Furthermore, even among public health proponents this communication style is believed to elicit, at best, short-term compliance rather than fostering genuine buy-in to public health recommendations (40).

The prevailing polarized climate fueled tensions between public health actors and the Florida government during the pandemic. Within this context, study participants pointed to how government may begin to repair negative perceptions tied with public health activities, emphasizing the value of transparency and a united front. Their calls to avoid censorship of public health officials and to establish consistency between government and public health messaging reveal a contentious reality that unfolded during Florida’s COVID-19 response. The cessation of daily reporting on the state COVID-19 dashboard (42) and the release of the advisory against vaccines for children (6) elicited considerable public criticism for FDOH. Additionally, the censoring of the former Florida Surgeon General following his promotion of masks likely reinforced distrust in public health institutions and validated the perception among some that proponents of such interventions are not credible. South Florida’s Sun-Sentinel echoed our findings, reporting on the silencing of FDOH personnel (43), despite their role as the public face for health guidance. Therefore, while key decisions had been made at the state level during the pandemic, Florida’s public health officials often carried the brunt of the public’s dissatisfaction. This reflects a concerning dynamic—the state governor retained decision-making power while public health officials bore the responsibility when public health actions clashed with the preferences of Florida’s political majority.

Participant responses underscored that trust should be mutual—to earn confidence, public health professionals and government should also be willing to place trust in the people they serve. That includes extending trust across all segments of the currently polarized public. Improved transparency and education that promotes scientific literacy can support building an informed public, strengthening the likelihood that trust—supported by buy-in rather than injunction—flows in all directions. In particular, encouraging science-based education emerged as a middle ground for participants who valued scientific evidence yet remained skeptical of the evidence around certain pandemic guidance. Honesty, open access to data, and formal science-based education provide individuals with the tools necessary to make data-driven decisions, which in turn, enables public health professionals to feel more confident in the public’s capacity to protect themselves. These calls to improve science-based education are indicative of the need for gradual norm change to promote voluntary adherence and sustainable buy-in to public health guidance rather than reliance on paternalistic imposition of policies.

Although political polarization is manifested in an apparent battle between individual freedom and collective good—with public health caught in the cross hairs— these concepts are actually deeply intertwined and highly valued by all Americans. Therefore, the notion of competing priorities does not have to be interpreted as a clash between individual rights and collective wellbeing. Instead, it can be viewed as encompassing divergent interpretations of foundational U.S. principles. Afterall, health, financial security, and autonomy are highly regarded by Americans as a whole. Our shared interests are exemplified by a Pew study, which found that Republicans and Democrats expressed similar levels of concern around the pandemic’s impact on the economy at 88 and 84%, respectively, (44). Moreover, our findings suggest that participants across the political spectrum often agreed on the challenges of cooperation and on broad objectives to overcome them, with divergence mostly occurring around why these obstacles had occurred.

American identity was built on the principle of “rugged individualism,” the interplay of a focus on self-reliance and a strong resistance to government interference (28). However, an undue emphasis on individualism overlooks the ways in which our cultural identity is shaped by mutual benefit—meaning that our idea of individual freedom cannot exist without community (27). All Americans, in fact, have come to rely on public institutions to provide collective protections for individuals, including Social Security, public education—and even public health, whose success is often invisible when there is no disease outbreak. Therefore, even in a society that prizes personal liberties and economic security, emphasis on these values does not rule out community, leaving space for public health efforts that depend on broad participation. Moreover, collective protection from health threats is necessary for individuals to exercise their autonomy and participate in a thriving economy. Despite political polarization obscuring our shared American identity, our core cultural values—individual freedoms and economic protections—can be leveraged to strengthen public health policy and practice. Through this understanding, we can identify potential pathways for rebuilding a public health system that reflects the values of individuals across the political spectrum without compromising the discipline’s foundational principles, fostering dialogue that is accessible, resonant, and avoids alienation.

Following a devastating pandemic, public health is in a moment of reckoning. *Can public health be reinvented and embraced once more as a trusted conduit of beneficence?* On the cusp of transformation, public health must seize this moment to reflect on what is within its control to change—acknowledging that several pandemic-era strategies inadvertently reinforced and widened divisions. Florida serves as a striking example of potential challenges that may lie ahead. With its centralized public health system, many FDOH professionals were restricted by the state with limited capacity to shape public health responses and recommendations during the pandemic. Recent developments—such as the dismissal of the CDC Director (45) and the U.S. Health Secretary’s sweeping changes to the vaccine landscape (46)—indicate that similar constraints on public health professionals are already taking shape nationwide. These challenges underscore the importance of identifying approaches that carefully consider feasibility and adapt to potential restrictions on public health policy and practice to strengthen cooperation.

To better advance its goal of collective wellbeing, we recommend a *cultural competency approach* to public health policies and practices, accounting for the diversity of cultures and values present in the U.S. In medicine, cultural competency—the provision of services that meet the sociocultural needs of diverse, often marginalized communities—has been known to improve utilization and patient/client health outcomes (47, 48). Cultural competency may be applied on a broader scale to shape public health’s engagement with the public, facilitating dialogue that acknowledges how different groups interpret and pursue the realization of our shared core values. Those interested in promoting public health should not take this macro-level cultural competency approach as one that disregards foundational public health principles, recognizing that values are deeply ingrained, and therefore not easily changed. Rather, it entails acknowledging prevailing societal values while fostering change through long-term initiatives and policies that are strategically communicated to avoid stigmatization and that resonate with the interests of the wider public. Therefore, to be culturally competent in public health policy and practice means an essential respect for all interests central to American identity, realizing that what is collectively valued, including personal autonomy and the economy, is fundamental to wellbeing.

A macro-level cultural competency approach does not require foregoing traditional public health practices, including vaccination and policy measures such as temporary restrictions and closures. Recognizing these interventions have demonstrated effectiveness during major pandemics like COVID-19 and 1918 influenza (49–53), this approach seeks to integrate knowledge of American cultural values to produce policies that are both well-received by the broader U.S. public and grounded in evidence-based research. Building on objectives suggested by study participants, we recommend the following considerations for public health professionals to incorporate cultural competence in our engagement with the public and policymakers in the context of infectious disease preparedness and response:

- To prevent stigmatization and polarization, avoid policies and messaging that convey judgement for nonadherence to public health guidance. Refrain from championing public health as a purely collectivist endeavor, which can alienate a segment of our population.
- Public health messaging that asks individuals to adopt self-limiting behaviors should be framed around practical, attainable actions (40) that validate individual agency and account for real-world constraints. Messaging should recognize different realities without using language that casts judgement for non-adherence to pressure individuals to cooperate.
- Tailor messaging based on what is known about a specific audience’s values and goals, fostering engagement through positive framing rather than divisive language. Public health messaging should appeal to the diversity of priorities, concerns, and values of different subgroups within the United States.
- Public health officials should seek collaboration with private sector and other relevant stakeholders to strengthen recommendations around pandemic restrictions. This may entail identifying which businesses would be affected, for how long, and under what conditions, to balance health protection with minimizing economic impacts.
- Public health professionals should emphasize their commitment to balancing safe practices with the timely resumption of commercial activity. To support this, they should consider the following ahead of future pandemics:

- Develop risk-based strategies to guide decisions around stay-at-home orders and the closures of schools and businesses.
- Create staged reopening plans, including operational toolkits and risk assessments.

## 5 Limitations

A detailed discussion of limitations is presented elsewhere (16). Specific to this analysis, potential limitations center on participant recruitment and candidness of responses. Although the study initially targeted county-level Florida Department of Health Directors and Administrators, our sample was later broadened to include other stakeholders in government, academia, and private sector due to challenges in recruitment. Recruitment of participants from local health departments was likely constrained by the state’s centralized response; some of our initial targeted sample declined to participate in the study, possibly because they lacked clearance from the state. The difficulty in securing sufficient participation to meet our intended aim may have also reflected Florida’s political landscape and the perceived risk associated with articulating views that conflicted with the state’s stance. Afterall, during interviews participants referenced the replacement of the State Surgeon General (8) and the county FDOH Director who was placed on leave after urging staff members to get vaccinated (54). Considering this, there may also be limitations related to the candor of participants working in the Florida government, particularly when responses could be perceived as criticism of the state. However, potential response bias is likely limited given the diversity of participants and their responses, including explicit critique of state and national actions. Although this analysis is limited to distrust and competing priorities, we recognize that additional factors may also influence cooperation with public health recommendations. Further, while this study’s scope is limited to Florida, its findings may be relevant to other states and to national-level public health policy and practice. Future research may explore similar themes of distrust and competing priorities in other states and settings.

## Data Availability

The data analyzed in this study is subject to the following licenses/restrictions: Due to the sensitive and political nature of the topics discussed, data is stored with restricted access in the University of Miami Institutional Data Repository, which is managed by the University of Miami Libraries. Data containing potentially identifying information, such as those related to specific roles and locations, have been removed from the data set. Requests to access these datasets should be directed to repository.library@miami.edu.
